# Preclinical evaluation of a novel triple-acting PIM/PI3K/mTOR inhibitor, IBL-302, in breast cancer

**DOI:** 10.1038/s41388-020-1202-y

**Published:** 2020-02-10

**Authors:** Sean P. Kennedy, Michael O’Neill, Darren Cunningham, Patrick G. Morris, Sinead Toomey, Carmen Blanco-Aparicio, Sonia Martinez, Joaquin Pastor, Alex J. Eustace, Bryan T. Hennessy

**Affiliations:** 1 Medical Oncology Group, Department of Molecular Medicine, Royal College of Surgeons Ireland, Smurfit Building Beaumont Hospital, Beaumont, Dublin Ireland; 2 Inflection Biosciences, Anglesea House, Blackrock, Dublin Ireland; 3 Cancer Clinical Trials and Research Unit, Beaumont Hospital, Dublin, Ireland; 4 Experimental Therapeutics Programme, Spanish National Cancer Research Centre (CNIO), Madrid, Spain; 5 Molecular Therapeutics for Cancer in Ireland, National Institute for Cellular Biotechnology, Dublin City University, Dublin, Ireland; 6 Cancer Trials Ireland, Innovation House, Old Finglas Road, Botanic, Dublin, Ireland

**Keywords:** Breast cancer, Drug development, Targeted therapies, Cancer therapeutic resistance

## Abstract

The proviral integration of Moloney virus (PIM) family of protein kinases are overexpressed in many haematological and solid tumours. PIM kinase expression is elevated in PI3K inhibitor-treated breast cancer samples, suggesting a major resistance pathway for PI3K inhibitors in breast cancer, potentially limiting their clinical utility. IBL-302 is a novel molecule that inhibits both PIM and PI3K/AKT/mTOR signalling. We thus evaluated the preclinical activity of IBL-302, in a range of breast cancer models. Our results demonstrate in vitro efficacy of IBL-302 in a range of breast cancer cell lines, including lines with acquired resistance to trastuzumab and lapatinib. IBL-302 demonstrated single-agent, anti-tumour efficacy in suppression of pAKT, pmTOR and pBAD in the SKBR-3, BT-474 and HCC-1954 HER2+/PIK3CA-mutated cell lines. We have also shown the in vivo single-agent efficacy of IBL-302 in the subcutaneous BT-474 and HCC-1954 xenograft model in BALB/c nude mice. The combination of trastuzumab and IBL-302 significantly increased the anti-proliferative effect in HER2+ breast cancer cell line, and matched trastuzumab-resistant line, relative to testing either drug alone. We thus believe that the novel PIM and PI3K/mTOR inhibitor, IBL-302, represents an exciting new potential treatment option for breast cancer, and that it should be considered for clinical investigation.

## Introduction

The PIM (proviral integration of Moloney virus) family of serine/threonine protein kinases were first discovered in experimental lymphomas [1]. The PIM kinase family is made up of three members; PIM 1, PIM 2 and PIM 3 [2]. Functions of the PIM kinase family vary, from cell cycle regulation, proliferation, apoptosis, invasion, metastasis and senescence [3, 4]. PIM has been found to be overexpressed in clear-cell renal-cell carcinoma [5], head and neck carcinomas [6], prostate [7], testicular tumours [8] and several haematological and solid tumours [8–11]. Due to the potentially important role of the PIM kinase family in regulatory signalling pathways and increased expression levels across a variety of solid tumours, they have become attractive targets for therapeutic inhibition.

In recent years, there has been a growing number of publications highlighting the association of increased PIM 1 expression with poor clinical outcome in breast cancer [12–14]. The PIM kinase family has been indicated in sustaining activity of the PI3K/AKT/mTOR pathway through overlapping mechanisms [15]. The PI3K/AKT/mTOR signalling pathway is integral to many aspects of regular cellular function, such as cell growth and survival. This signalling pathway is known to play a critical role in key aspects of carcinogenesis including increased genomic instability, decreased apoptosis, increased cellular proliferation and aberrant cytoskeleton alterations [16, 17].

There are overlapping mechanisms in the PIM and PI3K pathway, which ultimately regulate mTOR activation [18]. PIM kinases can attenuate pro-apoptotic signals such as BAD, p27 and p21 overlapping the PI3K pathway. PIM kinases can also regulate growth through continued activation of mTOR in parallel with the PI3K pathway [19]. Upregulation of PIM 1 expression has been shown in prostate cancer following inhibition of AKT [20], providing further evidence for the idea of crosstalk between both PIM kinase and PI3K/AKT/mTOR pathways [21]. Inhibition of AKT can also induce increased expression of several different receptor tyrosine kinases such as HER2 through PIM 1-mediated regulation [2, 22].

IBL-302 is a first-in-class oral kinase inhibitor rationally designed to uniquely combine pan-PIM kinase, pan-PI3K and mTOR inhibition in a single agent. Currently, there are a number of different preclinical and phase I trials focused on PIM kinase inhibition [21, 23–29]; however, few have been successful, with a narrow therapeutic window, which resulted in a dose-limiting cardiac QTc prolongation (SGI-1776) [24] (NCT00848601) or other unintended off-target effects, resulting in lack of observed effect (AZD1208) [30] (NCT01588548). However, in combination with other inhibitors, preclinical and phase I clinical trials suggest more tolerability and higher efficacy for PIM kinase inhibitors [31, 32]. This suggests that IBL-302 may demonstrate ability as an anticancer agent. Indeed, IBL-302 has already shown efficacy in non-small-cell lung cancer, a number of solid tumour cell lines, including both oesophageal and prostate cancer cell lines [33, 34], and is currently being tested in both EGFR inhibitor-resistant lung and haematological, in vivo models of cancer [35].

The objective of this pre-clinical evaluation is to comprehensively determine and characterise the anti-tumour efficacy of IBL-302, in breast cancer models. Furthermore, we aim to elucidate whether there is a synergistic effect of IBL-302 and trastuzumab in breast cancer models, including in vitro models of trastuzumab resistance, given the published role of PI3K/AKT/mTOR, and more recently of PIM kinase signalling in trastuzumab resistance [36].

## Results

### Effect of IBL-302 on breast cancer cell lines

**Fig. 1:**
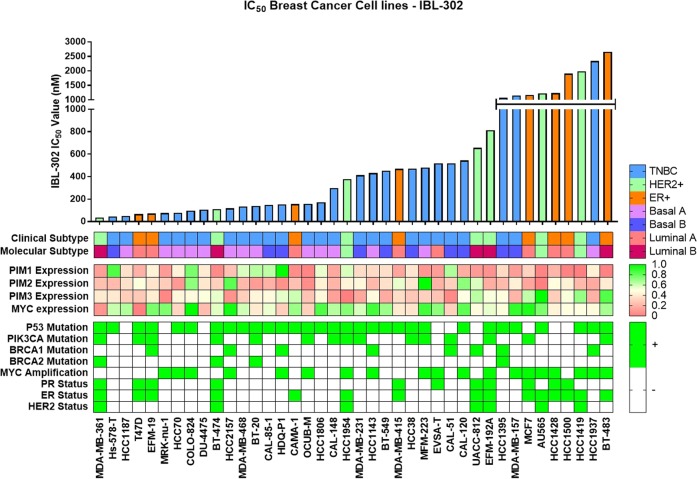
IBL-302 was tested in a panel of 40 breast cancer cell lines using CellTiter-Glo anti-proliferation assay (72 h of incubation). The panel of 40 breast cancer cell lines are subdivided into their clinical and molecular subtypes: HER+ (Green), ER+ (Orange) and TNBC (Blue). Each cell line’s corresponding PIM 1, PIM 2, PIM 3 and MYC expression levels, P53, PIK3CA, BRCA1 and BRCA2 mutation status, MYC amplification status and ER and PR status are displayed in Table 1.

IBL-302 GI_50_ values were determined via CellTiter-Glo anti-proliferation assay in a panel of 40 breast cancer cell lines. In Fig. 1 these cell lines are subdivided into their clinical and molecular subtypes, then ordered according to their GI_50_ value. The GI_50_ values for IBL-302 in the breast cancer cell lines ranged from 36 nM (MDA-MB-361) to 2656 nM (BT-483). There appear to be three plateaus of drug sensitivity: one at <200 nM, one at 200–800 nM and one at >1000 nM. PIM 1, PIM 2 and PIM 3 mRNA expression values in each of the 40 breast cancer cell lines are also shown in Fig. 1.

**Table 1:** Determination of the average IBL-302 IC_50_ value for each of the breast cancer cell lines listed after 120-h incubation. Each cell line’s corresponding PIM 1, PIM 2, PIM 3 and MYC expression levels, PIK3CA and PTEN mutation status, MYC amplification status, and ER and PR status are displayed in the table.

IBL-302 single-agent expression data/statistics										
Cell line ID	IBL-302 IC_50_ (nM)	MYC amplification	MYC expression	PR+	ER+	PTEN mutation	PIM 1 expression	PIM 2 expression	PIM 3 expression	PI3K mutation
MDA-MB-361	37	No	8.803	Yes	Yes	No	3.57	3.201	8.154	Yes
Hs-578-T	45	No	10.865	No	No	No	6.133	3.172	8.128	No
HCC1187	49	No	11.031	No	No	No	4.453	3.377	7.708	No
T47D	65	No	8.512	Yes	Yes	No	3.803	3.586	7.657	Yes
EFM-19	70	No	11.42	Yes	Yes	No	4.682	3.396	8.206	No
MRK-nu-1	75	Yes	11.391	No	No	No	3.516	3.266	6.715	No
HCC70	79	Yes	9.758	No	No	Yes	4.686	2.983	8.332	No
COLO-824	96	Yes	12.354	No	No	No	5.843	3.884	8.376	No
DU-4475	106	No	9.401	No	No	No	4.604	3.413	8.591	No
BT-474	110	No	10.685	Yes	Yes	No	3.302	3.289	8.723	Yes
HCC2157	117	Yes	12.205	No	No	No	4.045	4.019	6.855	No
MDA-MB-468	134	No	10.81	No	No	Yes	5.204	3.16	7.738	No
BT-20	138	No	10.056	No	No	No	5.441	3.002	8.309	Yes
CAL-85-1	148	No	10.601	No	No	No	5.391	3.114	8.383	No
HDQ-P1	154	Yes	9.654	No	No	No	6.697	2.947	8.747	No
CAMA-1	155	No	11.199	No	Yes	Yes	3.347	3.195	8.94	No
OCUB-M	157	Yes	9.677	No	No	No	3.234	2.924	7.571	Yes
HCC1806	171	No	11.761	No	No	No	5.1	3.461	8.351	No
CAL-148	299	No	11.005	No	No	Yes	3.685	3.13	7.067	No
HCC-1954	379	Yes	11.339	No	Yes	No	5.215	3.479	6.596	Yes
MDA-MB-231	413	No	10.754	No	No	No	4.389	3.493	7.178	No
HCC1143	431	Yes	11.835	No	No	No	4.381	3.16	8.387	No
BT-549	454	No	11.172	No	No	Yes	4.434	3.374	7.225	No
MDA-MB-415	470	No	10.057	Yes	Yes	Yes	3.668	3.64	7.967	No
HCC38	471	No	9.959	No	No	No	4.524	3.404	7.62	No
MFM-223	481	Yes	11.836	No	No	No	3.457	4.279	7.767	Yes
EVSA-T	518	Yes	11.28	Yes	No	Yes	3.543	3.126	7.579	No
CAL-51	519	No	9.701	No	No	Yes	5.484	3.286	6.736	Yes
CAL-120	545	Yes	11.632	No	No	No	3.761	3.887	8.521	No
UACC-812	656	No	10.296	Yes	Yes	No	3.523	3.682	9.066	No
EFM-192A	813	Yes	10.626	Yes	Yes	No	3.409	3.096	8.612	Yes
HCC1395	1068	No	10.488	No	No	No	4.374	3.167	8.425	No
MDA-MB-157	1154	Yes	11.964	No	No	No	3.551	3.456	7.353	No
MCF7	1172	Yes	12.124	Yes	Yes	No	4.273	3.055	9.009	Yes
AU565	1216	Yes	11.405	No	Yes	No	3.223	2.852	10.46	No
HCC1428	1232	Yes	10.198	Yes	Yes	No	3.877	3.525	7.735	No
HCC1500	1903	No	10.375	Yes	Yes	No	3.199	3.46	8.598	No
HCC1419	1985	Yes	6.421	No	Yes	No	3.159	3.156	9.332	No
HCC1937	2343	Yes	10.301	No	No	No	4.664	3.081	7.309	No
BT-483	2657	Yes	11.105	No	Yes	No	3.188	3.436	9.829	Yes

**Fig. 2:**
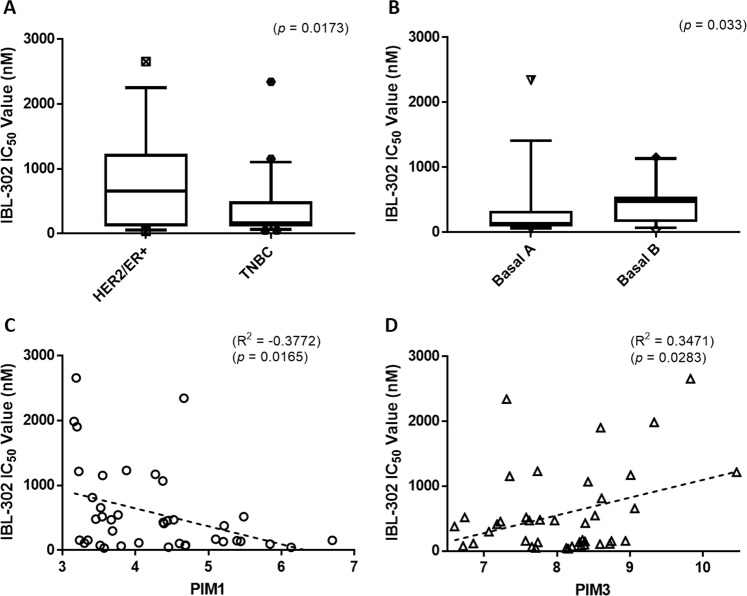
Using the clinical subtype, molecular subtype and PIM 1/3 expression values of 40 breast cancer cell lines, IBL-302 sensitivity (based on IC_50_) was examined. (**a**) Compares triple-negative versus HER2-amplified/ER+ breast cancer cell lines in terms of IBL-302 IC_50_ values. (**b**) Compares Basal A triple-negative with basal B triple-negative breast cancer cell lines in terms of IBL-302 IC_50_ values. (**c**) Compares PIM 1 expression across the 40 breast cancer cell lines with IBL-302 IC_50_ values and (**d**) compares PIM 3 expression across the 40 breast cancer cell lines with IBL-302 IC_50_ values.

Using the data in Fig. 1, we stratified the breast cancer cell lines based on their clinical subtype and mRNA expression profiles. We then examined their sensitivity to IBL-302 (Fig. 1). The results show that triple-negative breast cancer cell lines (TNBC) are more sensitive to IBL-302 (Fig. 2a) than HER2+/ER+ breast cancer cell lines (*p* < 0.017). TNBC cell lines express more PIM 1 (Supplementary Fig. 1) when compared with HER2+/ER+ subtypes (*p* < 0.017). When subcategorising the TNBC cell lines further into Basal A and Basal B, the Basal A breast cancer subtype showed a significantly increased sensitivity to IBL-302 (Fig. 2b) when compared with the Basal B subtype (*p* < 0.033). When comparing PIM 1 mRNA expression versus IBL-302 GI_50_ values (Fig. 2c), there was a significant inverse correlation; in other words, increased PIM 1 expression was correlated with lower IBL-302 GI_50_ values (*p* < 0.016). Conversely, when comparing PIM 3 mRNA expression versus IBL-302 GI_50_ values (Fig. 2d), there was a significant positive correlation; in other words, increased PIM 3 expression was correlated with increased IBL-302 GI_50_ values (*p* < 0.028). When the cell lines were divided via their therapeutic response to IBL-302 (responsive = <400 nM IBL-302 IC_50_ and non-responsive = > 400 nM IBL-302 IC_50_), in cell lines that were responsive to IBL-302, there was higher PIM 1 expression, lower PIM 3 expression and lower levels of MYC amplification (Supplementary Fig. 2A–C). IBL-302 sensitivity correlates with higher PIM 1 expression in HER2+/ER+ cell lines, but not in TNBC cell lines (Supplementary Fig. 2D–I).

### PIM/AKT/PI3K inhibition and induction of apoptosis in breast cancer cell lines

**Fig. 3:**
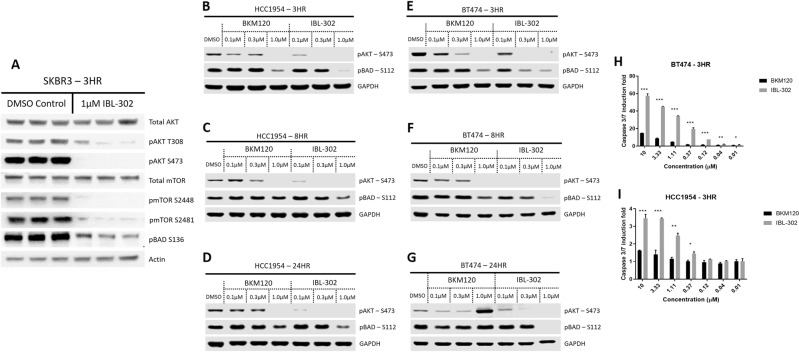
Impact of IBL-302 on PIM/AKT/PI3K signalling components and induction of apoptosis in breast cancer cells. **a** SKBR-3 cells were treated with DMSO and IBL-302 (1 µM) for 3 h. The resulting total AKT, pAKT-(S473), pAKT-(T308), total mTOR, pmTOR-(S2448), pmTOR-(2481) and pBAD-(S138). **b**–**g** BT-474 and HCC-1954 cells were treated with ascending doses of BKM120 and IBL-302 (0.1, 0.3 and 1 µM) for 3, 8 and 24 h. The resulting pAKT-(S473) and pBAD-(S112) were analysed via western blot analysis. **h**, **i** BT-474 and HCC-1954 cells were treated with increasing doses of BKM120 and IBL-302 (0–10 µM) for 3 h. The resulting caspase 3/7 induction was analysed via caspase assay.

To demonstrate that IBL-302 was inhibiting its intended targets (PIM/AKT/PI3K), western blot analysis was utilised. Inhibition of pAKT-(S473) and pAKT-(T308) was assessed as a measure of PI3K inhibition [37–42], while pBAD-(S112) and pBAD-(S136) were assessed as a measure of PIM kinase inhibition (pBAD-(S136)/(S112) were utilised as a readout of PIM kinase activity as there are no reliable, commercial pPIM kinase antibodies available) [27, 43]; pmTOR-(S2448) and pmTOR-(S2481) was also assessed as a measure of mTOR inhibition. Upon treatment with IBL-302 at 1 µM for 3 h, the levels of pAKT-(T308) (*p* = 0.049), pAKT-(s473) (*p* = 0.001), pmTOR-(S2448) (*p* = 0.001), pmTOR-(S2481) (*p* = 0.006) and pBAD-(S136) (*p* = 0.011) were all significantly reduced when compared with a DMSO control (Fig. 3a). Inhibition of both pAKTs, both pmTORs and pBAD highlights IBL-302's ability to inhibit the PI3K/mTOR and PIM pathways. Upon treatment with IBL-302 at (0.1, 0.3 and 1 μM) for (3, 8 and 24 h), the levels of pAKT-(S473) and pBAD-(S112) were reduced in BT-474 and HCC-1954 (Fig. 3b–g). The effect of IBL-302 on pAKT-S473 is observed at all concentrations in both BT-474 and HCC-1954, while only the highest concentration of BKM120 (pan-PI3K inhibitor) completely inhibits pAKT-(S473) phosphorylation. IBL-302 inhibited pBAD-(S112) at both 0.3 and 1 µM concentrations of IBL-302 in BT-474 and HCC-1954 at the 3- and 8-h timepoints (Fig. 3b–g).

To determine whether IBL-302 induced death via apoptosis, a caspase 3/7 induction assay was performed on both BT-474 and HCC-1954 cells for 3 h (Fig. 3h, i). There is a significant enhancement of apoptotic induction observed in Fig. 3h, i in both BT-474 and HCC-1954 over the 3-h incubation in both IBL-302 and BKM120 as the therapeutic concentration increases; however, IBL-302 induces significantly more activation of caspase 3/7 when compared with BKM120 at similar concentrations (BT-474 (*p* < 0.0.027) and HCC-1954 (*p* < 0.041)).

The apoptotic effect of IBL-302 was further analysed on BT-474 and HCC-1954 cell lines via dual staining with annexin V and propidium iodide. BT-474 and BT-474-T cell lines were treated with IBL-302 alone (1 µM), trastuzumab alone (10 mg/ml), or IBL-302+ trastuzumab (1 µM/10 mg/ml) for 72 h (Supplementary Figs. 3 and 4A, B). The results show that treatment with IBL-302 single agent or trastuzumab significantly induced cell apoptosis in BT-474-P, BT-474-T, HCC-1954-P and HCC-1954-L cell lines relative to untreated controls. The early apoptotic rates induced by the IBL-302 and trastuzumab combination was significantly higher than that of either IBL-302 or trastuzumab alone (Supplementary Figs. 3 and 4A, B) (BT-474-P (*p* < 0.003), BT-474-T (*p* < 0.001), HCC-1954-P (*p* < 0.001) and HCC-1954-L (*p* < 0.006)).

### Efficacy of IBL-302 in breast cancer subcutaneous xenograft mouse models

**Fig. 4:**
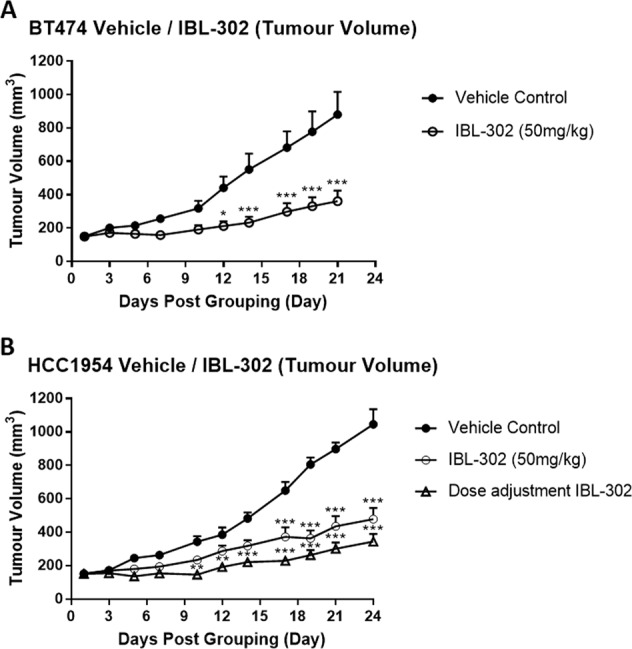
In vivo efficacy of IBL-302 was determined in BT-474 and HCC-1954 subcutaneous xenograft models in BALB/c nude mice. **a** BT-474 xenograft mice were treated with vehicle control and IBL-302 (50 mg/kg). BT-474 tumour (mean ± SE) volumes were measure and plotted as a function of time. *P* values were determined via Student *t* test and (*p* < 0.05) was considered to be statistically significant. **b** HCC-1954 xenograft mice were treated with vehicle control, IBL-302 (50 mg/kg) and dose adjustment IBL-302 (as described in ‘Materials and methods’ section). HCC-1954 tumour (mean ± SE) volumes were measured and plotted as a function of time. *P* values were determined via Student *t* test and (*p* < 0.05) was considered to be statistically significant.

To evaluate the in vivo efficacy of IBL-302, we chose two cell lines, BT-474 (from the most sensitive group in vitro) and HCC-1954 (from the group of intermediate in vitro sensitivity) from Fig. 1. We examined the effect of IBL-302 in vivo using BT-474 and HCC-1954 subcutaneous xenografts in BALB/c nude mice. The tumour volumes of the animals were plotted against time (days) (Fig. 4a, b). In the BT-474 xenograft study, in comparison with the vehicle control group, the IBL-302 (50 mg/kg) treated group demonstrated a highly significant reduction (*p* < 0.05) in the tumour volumes from day 12 to day 21 (Fig. 4a). There was an 8% bodyweight loss in the IBL-302- (50 mg/kg) treated group 10 days after the first treatment (Supplementary Fig. 5A), which was the reason for the introduction of the third treatment group in the xHCC1954 study. In this third group, vitamin E was included to prolong gut absorption of IBL-302, while the overall IBL-302 dose was reduced to improve gastrointestinal tolerability of treatment. In the xHCC1954 xenograft study, in comparison to the vehicle control group, the IBL-302 (50 mg/kg) treated group demonstrated a significant reduction (*p* < 0.05) in tumour volumes from day 17 to day 24 after treatment initiation (Fig. 4b). However again, there was an 8% bodyweight loss in the IBL-302 (50 mg/kg) treated group starting at 10 days post 1st treatment (Supplementary Fig. 5B). This weight loss effect was essentially reversed in the dose-adjusted IBL-302-treated group in the xHCC1954 study (Supplementary Fig. 5B) (as described in ‘Materials and methods’ section) without a deleterious effect on the anti-tumour efficacy of IBL-302 (Fig. 4b).

### IBL-302 efficacy in trastuzumab and lapatinib-resistant breast cancer cell lines

Given the published roles of PI3K/AKT/mTOR, and more recently of PIM kinase signalling in mediating resistance to HER2- targeted therapies [36], we evaluated IBL-302 in breast cancer models of acquired trastuzumab and lapatinib resistance. To examine the effect of IBL-302 used as a single agent, and in combination with trastuzumab, cell proliferation assays were employed in BT-474-P, BT-474-T, SKBR-3-P, SKBR-3-T, SKBR-3-L, HCC-1954-P and HCC-1954-L cell lines. These cell lines are defined in the methods with P representing the parental cell lines. IBL-302 performed well as a single agent in resistant cell lines, achieving GI_50_ values ranging from 33.4 nm ± 2.9 nm in SKBR-3-T cell line to 165.22 nm ± 17.1 nm in HHCC1954-L (Table 2).

**Table 2:** Determination of the average IBL-302 IC_50_ value for each of the breast cancer cell lines listed after 120-h incubation. Results are mean ± SD values for three independent experiments.

IBL-302 single-agent IC_50_ panel							
Cell line ID	SKBR-3-P	SKBR-3-T	SKBR-3-L	BT-474-P	BT-474-T	HCC-1954-P	HCC-1954-L
Average IC_50_ (nM)	76.1 ± 8.9	33.4 ± 2.9	136.6 ± 35.2	91.2 ± 2.1	66.1 ± 1.3	144.6 ± 8.9	165.2 ± 17.1

**Fig. 5:**
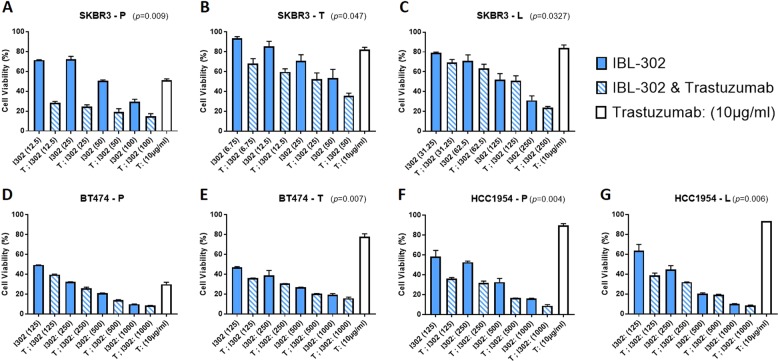
Acid phosphatase assay showing the efficacy of IBL-302 on viability in trastuzumab and lapatinib resistant breast cancer cells. **a** SKBR-3 trastuzumab-resistant (SKBR-3-T) and (**b**) SKBR-3 lapatinib-resistant (SKBR-3-L) cells. **c** BT-474 parental (BT-474-P) and (**d**) BT-474 trastuzumab resistant (BT-474-T). **e** HCC-1954 parental (HCC-1954-P) and (**f**) HCC-1954 lapatinib-resistant (HCC-1954-L) (**g**) cells after the combination of ascending amounts of IBL-302 (nM) with a static amount of trastuzumab (10 µg/ml). Significance was determined via Kruskal–Wallis, non-parametric test through utilisation of Prism software (* > 0.05) (** > 0.01) (*** > 0.001).

Enhancement of the anti-proliferative efficacy was observed when IBL-302 and trastuzumab were combined across the seven breast cancer cell lines (Fig. 5a–g). In SKBR-3-P, SKBR-3-T and SSKBR3L cells, the combination of IBL-302 and trastuzumab was significantly more effective than either single agent alone (Fig. 5a–c). It was shown in SKBR-3-T cell line that 50 nM IBL-302 10 mg/ml trastuzumab combination was significantly more effective than either therapeutic alone (*p* = 0.002) (Fig. 5b). The trastuzumab sensitive BBT474-P and the resistant BBT474-T cell lines both showed significantly more growth inhibition with the IBL-302 and trastuzumab combination when compared with each agent alone (Fig. 5d, e). Lastly, the intrinsically trastuzumab-resistant HCC-1954-P and HCC-1954-L cell lines both showed significantly more growth inhibition with the combination of IBL-302 and trastuzumab compared with each therapeutic alone at most evaluated doses (Fig. 5f, g).

### Western blot analysis of IBL-302-treated BT-474 xenograft tumours

Western blot analysis was also performed on tumour samples obtained from xenograft in vivo models of BT-474 8 h and 24 h post the last dose with BKM120 (35 mg/kg) and IBL-302 (50 mg/kg). The effect of IBL-302 on pAKT-(S473), pBAD-(S112) and PIM 1 was not significant when compared with either the vehicle control or BKM120-treated tumour samples (Supplementary Fig. 6).

## Discussion

PIM and PI3K kinases can mediate several of their cellular functions through subsequent phosphorylation of a variety of intersecting downstream signalling components. They are crucial to several cellular processes, including genomic instability (e.g., through an interaction between PIM 1 and NUMA [7, 44]), cell cycle regulation (e.g., through PIM 1 phosphorylation of p21waf1 [45, 46]) and induction of senescence (e.g., through overexpression of PIM 1 [47–49]). Considering the overlapping effect of these kinases on normal and cancerous cellular function, an inhibitor that targets both the PIM and the PI3K/AKT/mTOR pathway would have potential utility in cancer treatment.

In this study, the pan-PIM, PI3K and mTOR inhibitor, IBL-302, demonstrated efficacy across 40 breast cancer cell lines with GI_50_s ranging from 36 nM (MDA-MB-361) to 2656 nM (BT-483). IBL-302 has been shown to inhibit PI3Kα (2.08 nM) to roughly the same degree of efficacy as PIM 2 (7.74 nM) and PIM 3 (5.86 nM), while IBL-302 has been shown to have lower efficacy against mTOR (81.3 nM) and PIM 1 (22.8 nM) [50]. Stratifying the 40 breast cancer cell lines into subtype showed that TNBC cell lines were most sensitive to IBL-302 (*p* < 0.017) (Fig. 2a). The TNBC cell lines also had significantly increased levels of PIM 1 when compared with the HER2+ and ER+ cell lines (*p* < 0.01) (Supplementary Fig. 1). This has already been described by Brasó-Maristany et al. and others who identified increased PIM 1 expression in TNBC that correlated with increased proliferation and protection from apoptosis [12, 51].

When the 40 breast cancer cell lines were stratified via their PIM 1 expression, an increased PIM 1 expression level was correlated (*p* < 0.016) (Fig. 2c) with decreased IBL-302 GI_50_ value, while an increased PIM 3 expression level was correlated (*p* < 0.028) (Fig. 2d) with increased IBL-302 GI_50_ values. When the 40 breast cancer cell lines were stratified into HER2+/ER+ and TNBC groups and then stratified again via their therapeutic response to IBL-302, it was shown in the responsive groups that PIM 1 expression was elevated, PIM 3 expression was decreased and MYC amplification significantly reduced when compared with the non-responsive groups (Supplementary Fig. 2A–C). These findings suggest that IBL-302 sensitivity in cell lines, may be differentially affected by PIM 1 expression versus PIM 3 expression. There have been studies to suggest that PIM 3 plays an overall protective role in cells via protection against apoptosis and increased proliferation [52]. Increased levels of PIM 3 have also been previously shown to indicate poor prognosis and indicate minimal response to chemotherapy [53]. The roles of expression PIM 1 as predictors of response to IBL-302 require further exploration. The increased expression of PIM 1 in both TNBC and HER2+ cell lines, along with the correlation between IBL-302 sensitivity and elevated PIM 1 expression, also suggests the potential for further study of IBL-302 in TNBC and HER2+ cell lines.

Inhibition of PI3K/AKT can promote increased expression of both PIM 1 [20] and HER2 [22] among other compensatory pathways that can mediate resistance to PI3K/AKT inhibitors [54–56]. Other studies have demonstrated that PIM 1–3 are able to inhibit BAD-induced apoptosis and prevent therapeutic rescue [36]. This provides further rationale for the combination of the dual PI3K/PIM inhibitor IBL-302 with trastuzumab in an effort to overcome trastuzumab resistance. The synergistic, anti-proliferative effect that we have demonstrated of IBL-302 in combination with trastuzumab, across 7 HER2+ cell lines supports this rationale (Fig. 5a–g). This combinatorial effect of IBL-302 and trastuzumab was also prevalent in apoptotic readout when comparing both BT-474-P and BT-474-T cell lines (Supplementary Fig. 3A, B) and in HCC-1954-P and HCC-1954-L cell lines (Supplementary Fig. 4A, B). Our in vivo assessment of IBL-302 in subcutaneous xenograft models also shows that the PIM/PI3K/mTOR inhibitor, IBL-302, is effective in in vivo models of HER2+ breast cancer. Others have also shown that the targeting of PIM kinases with pan-PIM inhibitors can resensitise resistant models of disease, to other therapeutics [12, 57]. These data reinforce the current consensus, that the role of targeting PIM kinases to rescue sensitivity to some anticancer therapies should be further explored [57–59].

In this study, we have determined that elevated expression of PIM 1 is significantly associated with elevated sensitivity to IBL-302 in both TNBC and HER2+/ER+ cell lines. PIM 1 has been highlighted as a therapeutic target in TNBC [12, 14, 60] and as a result, not much focus has been placed on HER2+/ER+ cells, especially those with acquired resistance to HER2 targeted therapies. Interestingly, there is high PIM 1 expression in the HER2+ HCC-1954 cells, whilst the PIK3CA mutant BT-474 cells, which have low expression of PIM 1, are also sensitive to IBL-302. IBL-302 is a (PIM/PI3K/mTOR) inhibitor, so it maintains the potential to be therapeutically active in a cell line with high PIM 1 (like HCC-1954) and alternatively a cell line with mutant PIK3CA (BT-474) giving it a wider scope of use.

In recent studies with Copanlisib (a pan-PI3K inhibitor), it has been shown to be effective in advanced models of HER2+ breast cancer [61, 62]. Copanlisib has also demonstrated a preclinical and clinical benefit in women with trastuzumab refractor- advanced HER2+ breast cancer [61, 62]. The PI3K inhibitors utilised in these studies showed a cytostatic and not a cytotoxic effect. IBL-302 has demonstrated an ability to induce apoptosis individually and when combined with trastuzumab, potentially overcoming the limitation of current PI3K inhibitors (Fig. 3) (Supplementary Figs. 3, 4).

As referenced to in the introduction, there are several preclinical single-agent PIM kinase inhibitors available, but only a few have proceeded to phase I clinical trials, and been unsuccessful due to either a dose-limiting cardiac QTc prolongation (SGI-1776) [24] (NCT00848601) or lack of observed effect (AZD1208 in AML) [30, 57] (NCT01489722). As discussed earlier, PIM inhibitors in combination with another therapy may offer more promise. The pan-PIM kinase inhibitor, PIM447, is being tested in combination with Ruxotilinib, a JAK1/2 inhibitor in myelofibrosis [63] (NCT02370706). PIM447 (a pan-PIM kinase inhibitor) is being tested with BYL719 (a selective PI3Kα inhibitor) in multiple myeloma (NCT02144038). AZD1897 (pan-PIM kinase inhibitor) and AZD5363 (AKT inhibitor) in combination are being investigated in leukaemia [31, 32]. Thus, we have chosen to evaluate the preclinical efficacy of the novel dual PIM and PI3K/mTOR inhibitor, IBL-302 in breast cancer as described herein.

Overall, considering the encouraging evidence surrounding PIM kinase inhibitors in combination with other inhibitors in cancer therapy [31, 32, 63], and evidence suggesting targeting multiple pathways simultaneously will rescue sensitivity [18, 20, 22, 51], as well as the in vitro and in vivo studies of IBL-302 in preclinical breast cancer models presented in this paper, we believe that the dual PI3K/mTOR and PIM inhibitor IBL-302 should be investigated further in breast cancer. We thus plan, to take the compound to phase I clinical trial evaluation in heavily pre-treated breast cancer patients.

## Materials and methods

### CellTiter-Glo assay

The CellTiter-Glo screen was performed by Genomics of Drug Sensitivity in Cancer (GDSC) in the Wellcome Trust Sanger Institute, Hinxton UK. The drug screen took place over 2 equivalent sets of GDSC cell lines with 40 cells lines in total. The cell lines were seeded into 1536 well plates using an XRD-384 (Fluid-X) reagent dispenser. The number of cells seeded has been individually optimised for each cell line. IBL-302 was screened using a 7-pt dose response curve and a half-log (3.16-fold) dilution series. The dosing of the compounds was carried out using an ECHO 555 (Labcyte) acoustic dispenser, and the duration of drug treatment is 72 h. Cell number was measured using CellTiter-Glo (Promega) reagent. Cell lines were screened in RPMI-1640 media (Sigma) supplemented with 10% FCS and DMEM/F12 media (Sigma) supplemented with 10% FCS, both maintained at 37 °C with 5% CO_2_.

### Cell culture

Human HER2-amplified breast cancer cell lines were obtained from the Dublin City University (National Institute for Cellular Biotechnology) (DCU). Resistant variants were developed by continuous growth in relevant drug (SKBR-3 trastuzumab resistant (SKBR-3-T) (DCU): 100 µg/ml trastuzumab; SKBR-3 lapatinib resistant (SKBR-3-L) (DCU): 1000 nM lapatinib; HCC-1954 lapatinib resistant (HCC-1954-L) (DCU): 1000 nM lapatinib; BT-474 trastuzumab resistant (BT-474-T) (DCU): 100 µg/ml trastuzumab) for 6 months, with drug refreshed twice weekly [64, 65]. All cell lines were grown in RPMI-1640 media (Sigma) supplemented with 10% FCS and maintained at 37 °C with 5% CO_2_. Cell line identity was confirmed by DNA fingerprinting, which was performed by Source Biosciences™. Cell lines were Mycoplasma tested before and after the in vitro experiments. trastuzumab (21 mg/ml) was obtained from Beaumont Hospital and prepared in bacteriostatic water. IBL-302 was supplied by Inflection Biosciences and stock solutions prepared in dimethyl sulfoxide (DMSO) at 10 mM concentrations. BKM120 was supplied by Inflection Biosciences and stock solutions prepared in DMSO at 10 mM concentrations.

### Western blot analysis

To study the effect of IBL-302 on PI3K/AKT/mTOR and PIM kinase signalling, two breast cancer cell lines (BT-474 and HCC-1954) were treated with 0.1, 0.3 and 1 μM IBL-302 or BKM120 for 3, 8 and 24 h. SKBR-3 cells were also utilised for western blot analysis, and treated with 1 µM IBL-302 for 3 h. BT-474 and HCC-1954 Cells were treated and then lysed with 1× complete lysis buffer for 15 min at 4 °C and the lysate was then centrifuged at 13,000 rpm for 20 min at 4 °C. Supernatant was collected for protein quantification using a BCA kit. A total of 20 μg of sample per lane was loaded to a NuPAGE Novex 4–12% Bis-Tris protein gel and resolved. The protein was then transferred using the Invitrogen iBlot 2 gel transfer device and the nitrocellulose transfer stacks. The membrane was blocked for 1 h in 5% BSA at room temperature. The membrane was then incubated in primary antibodies (pAKT)-(S473) (CST, Cat No. 4060), pBAD-(S112) (CST, Cat No. 5284) and (GAPDH (Millipore, Cat No. MAB374) 1:5000) overnight at 4 °C. The membrane was then washed thrice and incubated with secondary antibodies (LI-COR Biosciences, Cat No. 926-32213 and Cat No. 926-68072) (1:10,000 dilution) for 1 h at room temperature. Image was captured on Li-Cor Odyssey imager and proteins were quantified. SKBR-3 cell lines were treated with 1 µM IBL-302 for 3 h and cells were treated and then lysed with 1× complete lysis buffer for 15 min at 4 °C and the lysate was then centrifuged at 15,000 rpm for 10 min at 4 °C. Supernatant was collected and protein quantified using a BCA assay. A total of 20 μg of sample per lane was loaded to a mini-PROTEAN® TGX 4–15% Bis-Tris protein gel and resolved. The protein was then transferred using the mini-PROTEAN® three-chamber wet transfer device and the nitrocellulose transfer stacks. The membrane was blocked for 1 h in 5% BSA at room temperature. The membrane was then incubated in primary antibodies (AKT) (CST, Cat No. 9272 S), (pAKT-T308) (CST, Cat No. 9275 S), (pAKT-S473) (CST, Cat No. 4060), (mTOR) (CST, Cat No. 2972 S), (pmTOR-S2448) (CST, Cat No. 2971 S), (pBAD)-(S136) (CST, Cat No. 9295) and (actin) (Sigma, Cat No. A5316) at a 1:1000 dilution overnight at 4 °C. The membrane was then washed thrice and incubated with secondary antibodies (1:5000 dilution) for 1 h at room temperature. Image was captured on Li-Cor Odyssey imager and proteins were quantified. Western blot densitometry measurements were obtained via imageJ analysis and significance was determined using a student’s *t* test, as compared with the untreated DMSO controls. (* ≤ 0.05) (** ≤ 0.01) (*** ≤ 0.001) (Supplementary Fig. 7).

### Apoptosis assays

To study the effect of IBL-302 on caspase 3/7 induction, two breast cancer cell lines (BT-474 and HCC-1954) were treated with increasing concentrations of IBL-302 (0.01–10 μM) for 4 h. Cells were seeded into a 96-well plate at 3000 cells per well for HCC-1954 and 12,000 cells per well for BT-474. Working solutions were prepared at 1:3 dilutions of 10 μM IBL-302 descending along ten points. The cells were seeded on day 1 and day 2, 0.5 µl of compound solution was transferred to each of the cell plate wells containing 100 µl of media. The plates were incubated with IBL-302 for 4 h at 5% CO_2_, 37 °C. The Caspase-Glo buffer was thawed by equilibrating to room temperature. The lyophilised Caspase-Glo 3/7 substrate was also equilibrated to room- temperature use. The Caspase-Glo 3/7 buffer and substrate were mixed to form the Caspase-Glo 3/7 reagent. After a 4-h IBL-302 incubation, addition of 100 µl of Caspase-Glo 3/7 Reagent is following by mixing on an orbital shaker for 10 min. The plate was allowed for 1 h to stabilise the luminescent signal and come to room temperature. The resulting luminescence was read at 500 nM on a plate reader.

To determine the stages of apoptosis (Early versus Late), both BT-474 and HCC-1954 cell lines, were treated with 1 µM IBL-302, 10 µg/ml trastuzumab and a combination of 1 µM IBL-302/10 µg/ml trastuzumab for 72 h. Cells were seeded into a six-well plate at 500,000 cells per well. The cells were seeded on day 1 and day 2, 1 µM IBL-302 was constituted in 1 ml of RPMI-1640, 10 ug/ml trastuzumab in 1 ml of RPMI-1640 and the combination of 1 µM IBL-302/ 10 ug/ml trastuzumab in 1 ml of RPMI-1640 and each corresponding wells media replaced with the treatment. After 72 h of treatment, the cells were detached using Trypsin, and washed twice with cold PBS before being exposed to Alexa Fluor^©^ 488 annexin V (5 µl) and 1 µl of 100 µg/ml propidium iodide diluted in 100 µl of annexin-binding buffer and incubated at room temperature for 20 min. After incubation cells were mixed with an additional 400 µl of annexin-binding buffer. The cells were then analysed via flow cytometry, measuring the fluorescence emission at 520 and 575 nm using a 488-nm excitation. Early apoptosis cell population were marked with Annexin V and the absence of propidium iodide (Q3), while late apoptosis was indicated by double staining of both annexin V and propidium iodide (Q2).

### Proliferation assays

For all resistant cell lines, drug was removed from the cells at least 7 days prior to starting assays, and no penicillin/streptomycin was added to media during proliferation assays; 5 × 10^4^ cells were seeded in 96-well plates. Plates are incubated overnight at 37 °C to allow cells to adhere. Therapeutics were added at the same time, to the plates at specific concentrations and incubated at 37 °C. Following 5-day incubation, during which control cells attained 80–90% confluence, all media was removed from the plates and washed once with PBS. Proliferation was measured using the acid phosphatase assay as previously described [66].

### In vivo anti-tumour efficacy

The in vivo animal experiments were carried out by ChemPartner^©^ company of Shanghai, China, and were composed of two independent studies of IBL-302. The first was a BT-474 xenograft model and second, a HCC-1954 xenograft model.

### BT-474 xenograft model

In the first study, 16 female nude mice (BALB/c nude mice), aged 6–8 weeks (about 18–20 g bodyweight), were implanted subcutaneously with a 0.5 mg Estradiol pellet 2 days prior to cell inoculation. The mice were inoculated subcutaneously at the right flank with xBT474 tumour cells (1 × 10^7^ cells/mouse) in 0.2-ml mixture of the base media with 50% matrigel to promote tumour growth. The animals were randomly divided into four groups with four in each group as follows; a vehicle control group (10% DMSO + 90% PEG400) 8 h and 24 h post the last dose, as well as an IBL-302 group (50 mg/kg) (10% DMSO + 90% PEG400) 8 h and 24 h post the last dose. Each of the groups was orally gavaged for the full 21 days at (50 mg/kg/day). The tumour size was measured three times a week, in two dimensions using a calliper and the volume expressed in mm^3^. The tumour volume was calculated using the formula: *V* = (0.5 × *A* × *B*^2^) where *A* and *B* were the long and short diameters of the tumour, respectively. The mice were sacrificed at the end of the experiment and fresh tumour tissues were collected.

### HCC-1954 xenograft model

In the second study, 24 female nude mice (BALB/c nude mice), aged 6–8 weeks (about 18–20 g of bodyweight), were implanted subcutaneously with a 0.5-mg Estradiol pellet 2 days prior to cell inoculation. The mice were inoculated subcutaneously at the right flank with xHCC1954 tumour cells (5 × 10^6^ cells/mouse) in 0.2 ml mixture of the base media with 50% matrigel to promote tumour growth. The animals were randomly divided into three groups with eight in each group as follows: a vehicle control group (10% DMSO + 90% PEG400), an IBL-302 group (50 mg/kg) (10% DMSO + 90% PEG400) and an IBL-302 group in a new formulation^1^ (10% DMSO + 70% PEG400 + 20% Vitamin E). Each of the groups was orally gavaged for the full 24 days at (50 mg/kg/day). The tumour size was measured three times a week, in two dimensions using a calliper and the volume expressed in mm^3^. The tumour volume was calculated using the formula: *V* = (0.5 × *A* × *B*^2^) where *A* and *B* were the long and short diameters of the tumour, respectively. The tumour size was then used for calculating tumour growth inhibition values. The mice were sacrificed at the end of the experiment and fresh tumour tissues were collected.

### Western blot analysis of xBT474 tumours

Western blot analysis was also performed on tumour samples obtained from xenograft in vivo models of BT-474 (xBT474). xBT474 (xenograft BT-474 tumour cells) mice were harvested at the end of the treatment, 8 and 24 h post the last dose with BKM120 (35 mg/kg) and IBL-302 (50 mg/kg). The tumour cells were treated and then lysed with 1× complete lysis buffer for 15 min at 4 °C, and the lysate was then centrifuged at 13,000 rpm for 20 min at 4 °C. Supernatant was collected and protein quantified using a BCA assay. A total of 20 μg of sample per lane was loaded to a NuPAGE Novex 4–12% Bis-Tris protein gel and resolved. The protein was then transferred using the Invitrogen iBlot 2 gel transfer device and the nitrocellulose transfer stacks. The membrane was blocked for 1 h in 5% BSA at room temperature. The membrane was then incubated in primary antibodies (pAKT-(S473) (CST, Cat No. 4060), pBAD-(S112) (CST, Cat No. 5284) and PIM 1 (CST, Cat No. 2907) at 1:1000 dilution) and (GAPDH (Millipore, Cat No. MAB374) 1:5000) overnight at 4 °C. The membrane was then washed thrice and incubated with secondary antibodies (1:10,000 dilution) for 1 h at room temperature. Image was captured on Li-Cor Odyssey imager and proteins were quantified.

### Statistical analysis

GI_50_ and effective dose (ED_50_) were calculated using CalcuSyn software (Biosoft). A students' two-tailed *t* test was used to compare the difference between TNBC and HER2+/ER+ groups when stratifying using the GI_50_ value. A students' two-tailed *t* test was also used to compare Basal A and Basal B subtypes of TNBC in terms of the GI_50_ value. A two-way ANOVA, with a post hoc test and *p*-values adjusted using Tukey’s honest significant difference, was employed to determine whether the combination of IBL-302 and trastuzumab is more effective at reducing proliferation rates, when compared with either single therapeutic alone (Fig. 5).

## Supplementary information

Supplementary Figure 1

Supplementary Figure 2

Supplementary Figure 3

Supplementary Figure 4

Supplementary Figure 5

Supplementary Figure 6

Supplementary Figure 7

## Footnotes

1 New Formulation guideline: The animals in HCC-1954 IBL-302 group with new formulation, dosed down to 35 mg/kg on D8–D10; mice stopped dosing on D11; mice dosed down to 25 mg/kg on D12–D13; mice dosed down to 10 mg/kg on D14–D20; mice were adjusted to 15 mg/kg on D21–D24 when bodyweight recovered to the baseline.
