# Supplementary figures and images for: Preclinical evaluation of a novel triple-acting PIM/PI3K/mTOR inhibitor, IBL-302, in breast cancer
Source: Oncogene. 2020 Feb 10;39(14):3028–40. doi: 10.1038/s41388-020-1202-y (PMC7118022; doi:10.1038/s41388-020-1202-y)

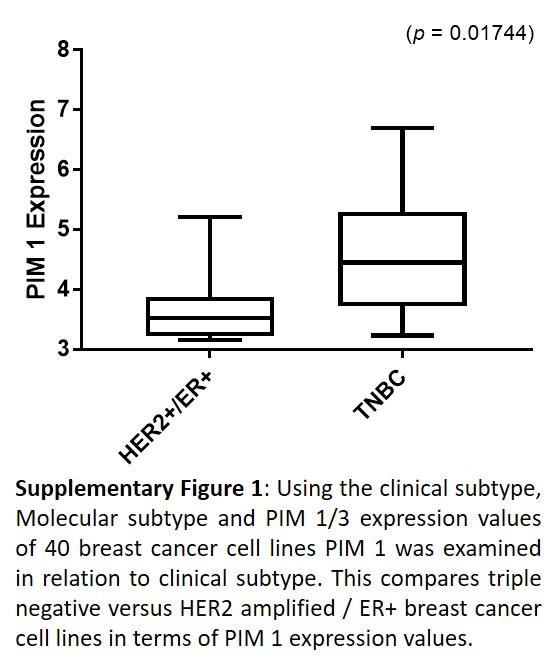

Supplement: Supplementary file 1 — Supplementary Figure 1 [file 41388_2020_1202_MOESM1_ESM.jpg]

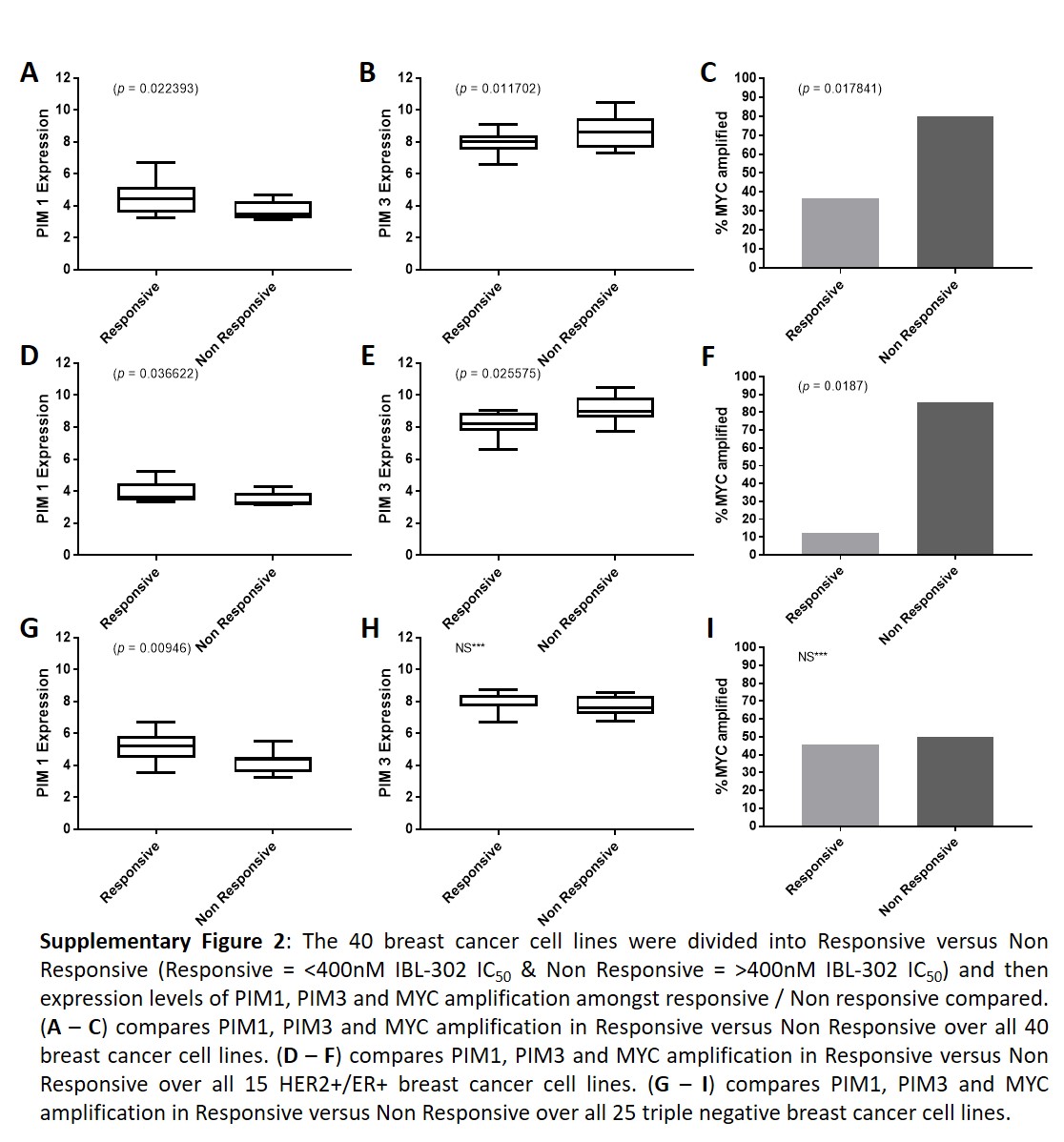

Supplement: Supplementary file 2 — Supplementary Figure 2 [file 41388_2020_1202_MOESM2_ESM.jpg]

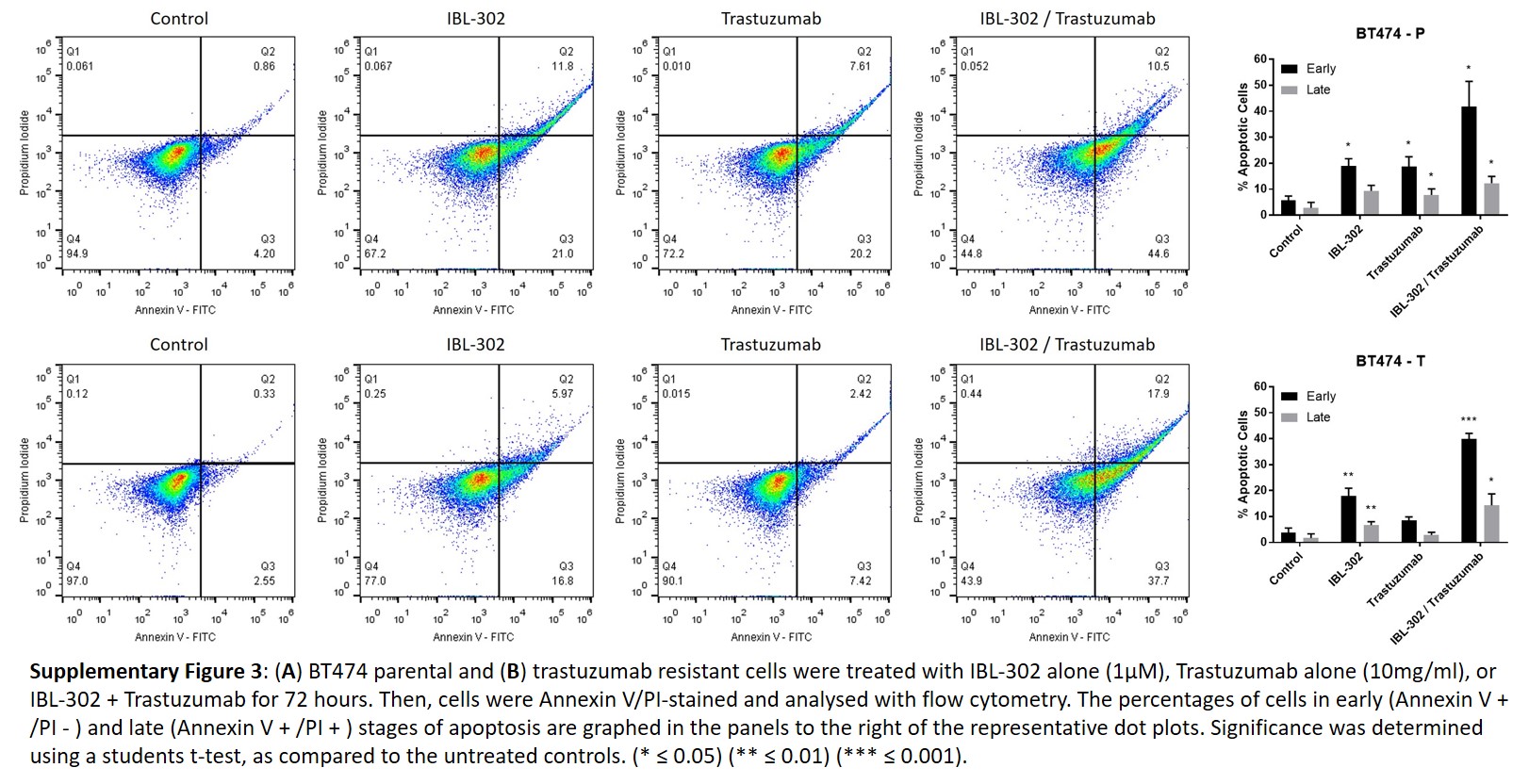

Supplement: Supplementary file 3 — Supplementary Figure 3 [file 41388_2020_1202_MOESM3_ESM.jpg]

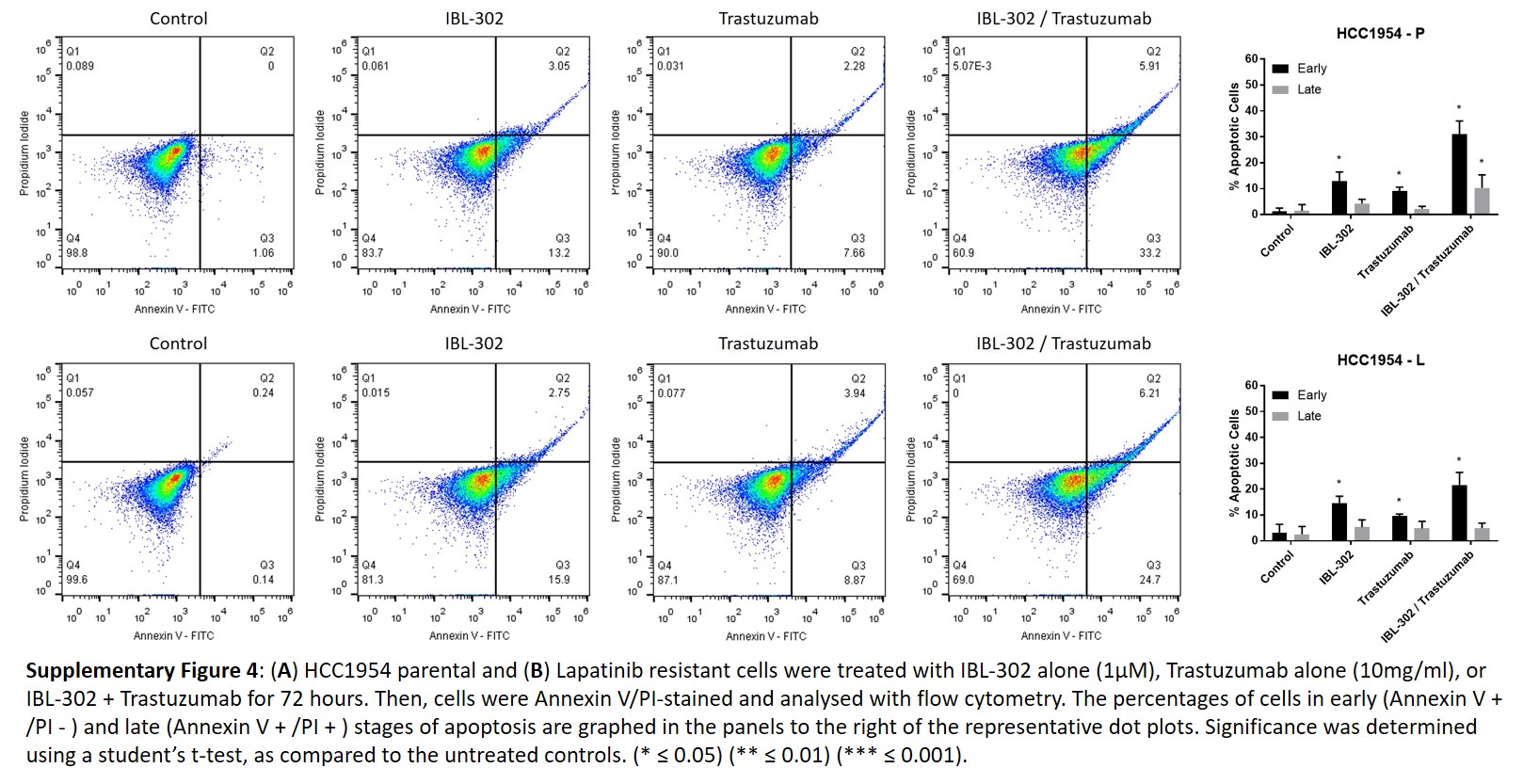

Supplement: Supplementary file 4 — Supplementary Figure 4 [file 41388_2020_1202_MOESM4_ESM.jpg]

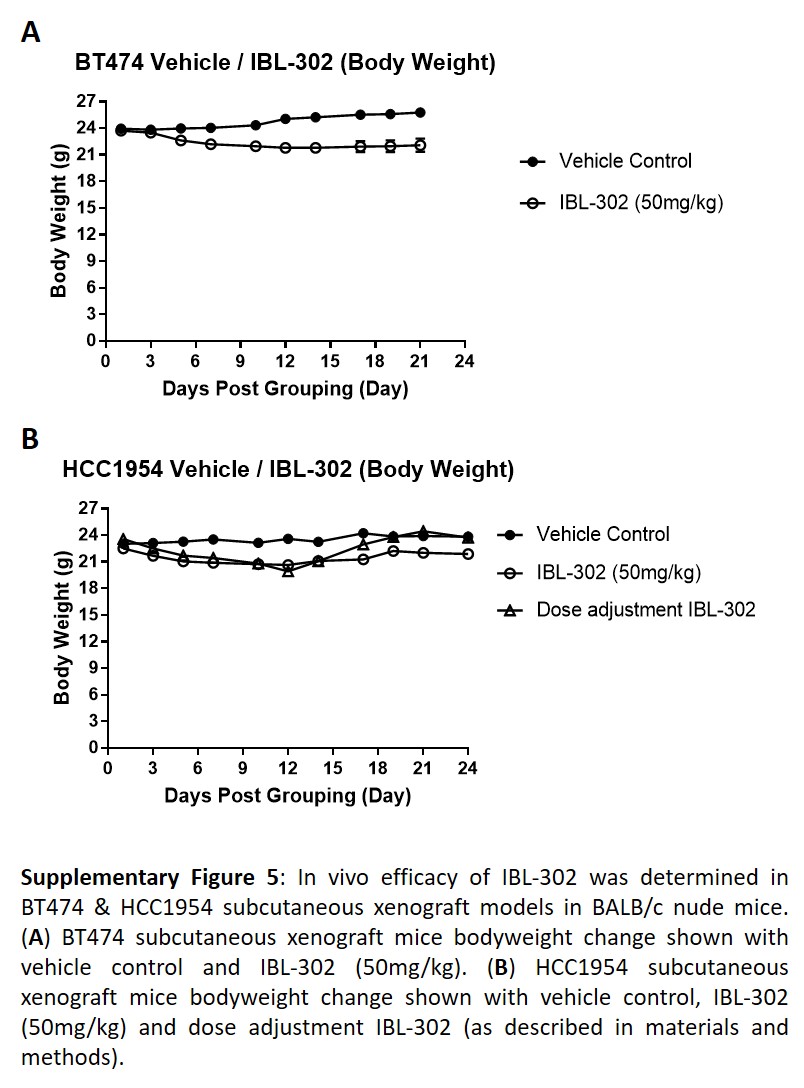

Supplement: Supplementary file 5 — Supplementary Figure 5 [file 41388_2020_1202_MOESM5_ESM.jpg]

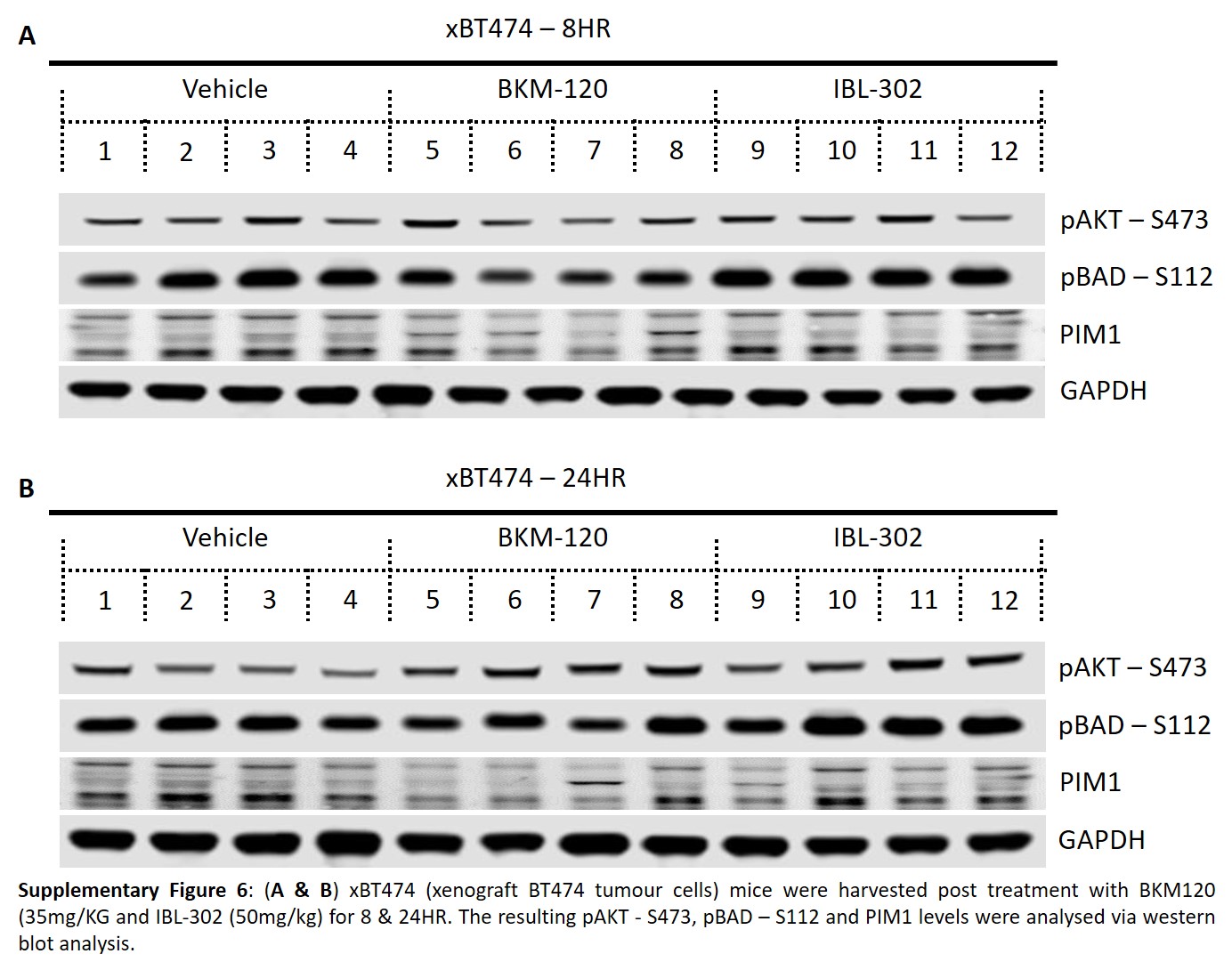

Supplement: Supplementary file 6 — Supplementary Figure 6 [file 41388_2020_1202_MOESM6_ESM.jpg]

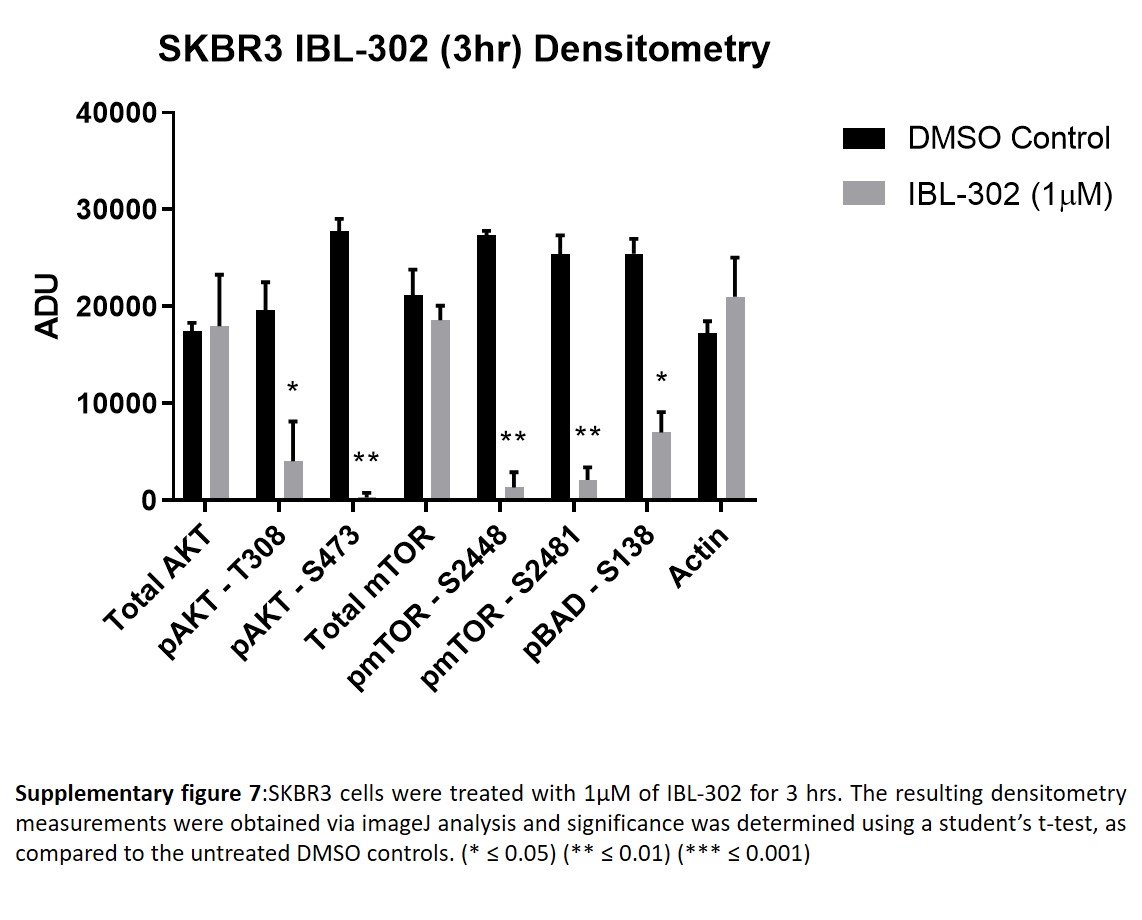

Supplement: Supplementary file 7 — Supplementary Figure 7 [file 41388_2020_1202_MOESM7_ESM.jpg]
